# In-vivo Imaging of Magnetic Fields Induced by Transcranial Direct Current Stimulation (tDCS) in Human Brain using MRI

**DOI:** 10.1038/srep34385

**Published:** 2016-10-04

**Authors:** Mayank V. Jog, Robert X. Smith, Kay Jann, Walter Dunn, Belen Lafon, Dennis Truong, Allan Wu, Lucas Parra, Marom Bikson, Danny J. J. Wang

**Affiliations:** 1 BioMedical Engineering Inter-Departmental Program (BME-IDP), University of California Los Angeles, Los Angeles, California, United States; 2 Laboratory of FMRI Technology (LOFT), Department of Neurology, University of California Los Angeles, Los Angeles, California, United States; 3 Stevens Neuroimaging and Informatics Institute, University of Southern California, Los Angeles, California, United States; 4 Department of Psychiatry, University of California Los Angeles, Los Angeles, California, United States; 5 Department of Biomedical Engineering, The City College of The City University of New York, New York, United States

## Abstract

Transcranial direct current stimulation (tDCS) is an emerging non-invasive neuromodulation technique that applies mA currents at the scalp to modulate cortical excitability. Here, we present a novel magnetic resonance imaging (MRI) technique, which detects magnetic fields induced by tDCS currents. This technique is based on Ampere’s law and exploits the linear relationship between direct current and induced magnetic fields. Following validation on a phantom with a known path of electric current and induced magnetic field, the proposed MRI technique was applied to a human limb (to demonstrate *in-vivo* feasibility using simple biological tissue) and human heads (to demonstrate feasibility in standard tDCS applications). The results show that the proposed technique detects tDCS induced magnetic fields as small as a nanotesla at millimeter spatial resolution. Through measurements of magnetic fields linearly proportional to the applied tDCS current, our approach opens a new avenue for direct *in-vivo* visualization of tDCS target engagement.

Transcranial direct current stimulation (tDCS) is a non-invasive neuromodulation technique that modulates cortical excitability with a small current (1–2 mA) applied through scalp electrodes. Since the demonstration of tDCS induced sustained changes in motor evoked potentials (MEP)^1^,^2^, there has been a steady increase of scientific reports on the neurophysiological, clinical and cognitive effects of tDCS. tDCS has been shown to improve symptoms in a wide range of neurologic and psychiatric disorders such as epilepsy, Parkinson’s disease, chronic pain, depression, drug cravings, and stroke (see review^3^,^4^). tDCS has also been shown to enhance learning, modulate working memory, and impart other cognitive benefits in healthy subjects^5^,^6^. Given its simple setup, high safety and low cost^7^, tDCS is emerging as a potential therapy as well as a tool for understanding the neurophysiology underlying various cognitive functions.

In order to understand the mechanisms of tDCS and improve its therapeutic potential, there is a critical need to identify, given a particular tDCS montage, (a) the brain areas that the applied current passes through, and (b) the neural circuits that it modulates (which may extend beyond the site of stimulation through brain networks). Attempts have been made using fMRI to detect hemodynamic changes (a surrogate marker for neuronal activity changes) resulting from tDCS. Results from blood oxygen level dependent (BOLD) fMRI indicate that tDCS elicits long-lasting, polarity dependent changes in BOLD signal and network connectivity during motor, visual and language tasks^8^,^9^,^10^,^11^ also reviewed in ref. 12. Polarity dependent changes in regional cerebral blood flow measured using arterial spin labeling (ASL) fMRI have also been reported, both during and following tDCS stimulation^13^. Additionally, electrophysiological changes due to tDCS stimulation have been recently measured in the brain using electroencephalogram (EEG) and magnetoencephalography (MEG)^14^,^15^,^16^.

In parallel to identifying neural circuits modulated by tDCS, computational modeling has been used to predict brain regions the tDCS current passes through or directly engages. Based on a finite element human head model^17^, this approach has been applied to derive the optimal electrode montage and dosage for tDCS^18^,^19^, as well as the design of ring electrodes with focal distribution of tDCS current^20^. However, the distribution of tDCS current is influenced by a host of interacting factors not fully accounted for by computational models. These include electrode geometry, electrical properties of tissue and cerebrospinal fluid (CSF), CSF flow, and complicated boundary conditions (due to the convoluted brain surface morphology). To date, estimates of tDCS current distribution remain theoretical and await experimental validation. In one approach, BOLD fMRI has been applied on post-mortem subjects to map tDCS effects without associated hemodynamic changes^21^. However, the authors note that imaging physics quantitatively linking the BOLD fMRI signal to current density has yet to be developed. In another study, voltage-recording probes were placed in the head of a cadaver to record the distribution of tDCS current^22^. However, this approach does not account for the effects of CSF flow and perfusion, as well as potential conductivity differences between cadaver and live tissue. At present, no *in-vivo* gold standard for mapping tDCS currents exists.

In this study, we present a novel MRI-based technique for direct visualization and quantification of the magnetic field changes induced by tDCS current. Detecting the magnetic field changes induced by tDCS is biologically significant since it can be applied as a marker to detect the current distribution and thereby the direct target engagement of tDCS. The proposed technique is based on Ampere’s law, which states that a direct current induces a linearly dependent magnetic field (Equation (1) in Methods). The induced magnetic field along the static field (*B*_*z*_) of the MRI can be detected using field mapping. Exploiting the linear relationship between applied current and induced magnetic field, a concurrent tDCS-MRI experiment was performed where magnetic field maps were acquired for a range of tDCS currents (0–1.5mA, applied in a pseudo random order). Post acquisition, the observed fields were fit to the applied current within the framework of a general linear model (GLM). The slope of such a linear fit can be interpreted as the tDCS-induced field (along *B*_*z*_) per unit applied-current with a confidence given by the goodness-of-fit.

The proposed MRI technique was first validated on a phantom where the current path and induced magnetic field was known. Subsequently, *in-vivo* feasibility in simple biological tissue was established with tDCS-MRI experiments on a human limb (calf), and the result was compared with a computational model of tDCS induced magnetic field changes. Finally, to demonstrate that this technique is practical for standard tDCS applications, the technique was applied to healthy subjects receiving tDCS with a bilateral motor cortices electrode montage. The results show that the proposed technique is able to detect magnetic field changes as small as a nanotesla (nT) with a spatial resolution of a few millimeters. Through measurements of magnetic fields that are linearly proportional to the applied tDCS currents (Ampere’s law), our approach opens a new avenue for direct visualization of tDCS target engagement. In contrast, existing experimental techniques use surrogate markers of the brain’s response (e.g., BOLD, ASL, EEG, MEG); measuring a secondary response to the applied tDCS which may extend beyond the site of stimulation due to brain networks.

## Results

### Phantom Experiment

**Figure 1:**
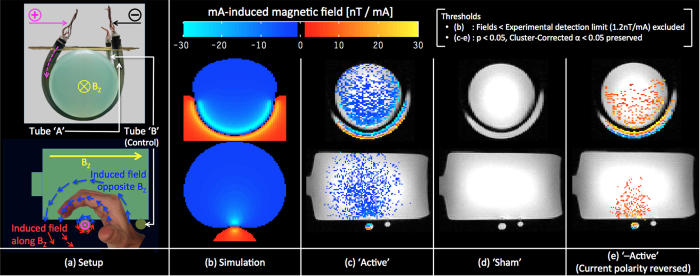
Setup and results of the Phantom experiment: (**a**) the phantom setup showing the induced magnetic fields intuited using Fleming’s right hand rule; (**b**) simulated magnetic field; (**c**–**e**) show experimentally detected current-induced magnetic fields from the ‘Active’, ‘Sham’ and ‘–Active’ sessions respectively. The experimental results were consistent with simulations (Cross-voxel correlations between the slices shown for simulated and {‘Active’; ‘–Active’} were {r = 0.84, p = 3.8 × 10^−267^, N = 989; r = −0.90, p = 2.3 × 10^−142^; N = 397}.

In order to validate our technique, we designed a phantom that channels the tDCS current into a known path. The current distribution was used to predict the current induced magnetic fields, which were compared with the experimentally measured fields. Our phantom consisted of a standard cylindrical MRI phantom with two U-shaped tubes (‘A’ and ‘B’ in Fig. 1a) wrapped around it. Both tubes contained identical electrolyte solutions and were made of plastic (a natural insulator). The ends of tube ‘A’ were connected to the tDCS stimulator, whereas the ends of tube ‘B’ were open. Consequently, all applied currents were confined to tube ‘A’. With such a configuration, magnetic fields induced by applied currents can be intuited using Fleming’s right hand rule (shown for the sagittal view in Fig. 1a). Tube ‘B’ served as a within-session ‘control’, carrying no applied current at any time.

Quantitative estimates of the current-induced magnetic field were computed using a finite element implementation of the Biot-Savart law (Fig. 1b), which were plotted alongside experimental results of the ‘Active’ session (Fig. 1c, thresholded at p < 0.05, Cluster corrected α < 0.05). As can be clearly seen, current induced magnetic fields detected by our technique are in close agreement with the simulation results (r = 0.84; p = 3.8 × 10^−267^; N = 989; cross-voxel correlation between slices shown in Fig. 1b,c). Data from a ‘Sham’ and ‘–Active’ session were also analyzed. The ‘Sham’ session was a control experiment where the tDCS stimulator was switched off during data acquisition. As expected, no significant current-induced fields were detected (Fig. 1d). In the ‘–Active’ session, the polarity of applied currents was reversed (compared to ‘Active’), which should result in a sign reversal of the induced magnetic fields while leaving the magnitudes intact (Ampere’s Law, Equation (1) in Methods). The detected current-induced magnetic fields in the ‘−Active’ session are consistent with this prediction (r = −0.90; p = 2.3 × 10^−142^; N = 397; cross-voxel correlation between slices shown in Fig. 1b,e).

**Figure 2:**
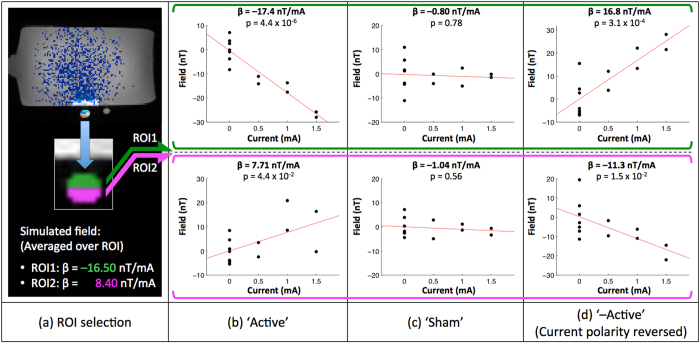
ROI analyses of the Phantom Experiment: Two ROIs from identical slices were selected (**a**) to show the average magnetic field vs. applied current. Simulated average fields in the ROIs were in good agreement with the experimentally measured fields induced by unit current in ‘Active’ and ‘-Active’ conditions (comparing slopes or β’s of Fig. 2a with **b, d** respectively). No significant fields were induced by currents in the ‘Sham’ condition (**c**).

Regions of interest (ROIs) consisting of the top (ROI1) and bottom (ROI2) halves of the current carrying ‘A’ tube (Fig. 2a) were selected from the same slice in all three sessions. Figure 2b–d show the average measured fields within the ROI as a function of applied current for the ‘Active’, ‘Sham’ and ‘–Active’ sessions respectively. Quantitatively, the measured fields per unit mA applied-current were found to be consistent with those calculated from simulations (by comparing slopes or β’s of Fig. 2b,d with a). The magnitude of estimated β’s varied from ~10–30nT/mA inside the current-carrying tube to 3–10 nT/mA inside the phantom. Qualitatively, the measured fields per unit mA applied-current were also consistent with the following four predictions: (1) For the same session (‘Active’ or ‘–Active’), the current-induced fields should have opposite signs between ROIs, as can be intuited using Fleming’s right hand rule (shown in Fig. 1a, Sagittal view). (2) For the same ROI, the current-induced fields should have opposite signs between ‘Active’ and ‘–Active’ sessions, as predicted by Ampere’s law (Equation (1) in Methods). (3) Current induced fields within ROI1 should be systematically higher than those of ROI2 because of the U-shaped geometry of the current carrying ‘A’ tube. Lastly, (4) there should be no detectable current-induced fields for the control ‘Sham’ session.

### Limb Experiment

**Figure 3:**
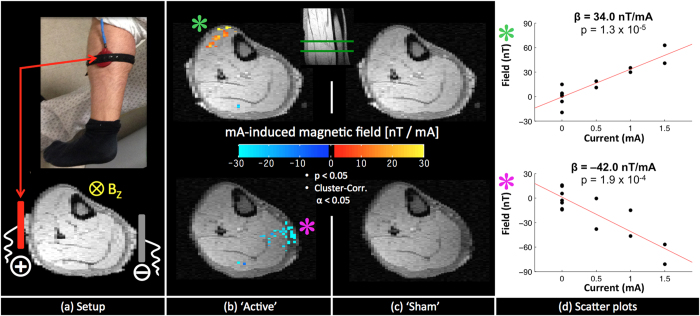
Setup and results of the Limb Experiment: (**a**) Limb experiment setup; (**b**) significant (p < 0.05, Cluster corrected α < 0.05) current-induced magnetic fields from the ‘Active’ session; No significant current-induced magnetic fields were found for the ‘Sham’ session (**c**,**d**) scatter plots of two significant clusters identified with an asterisk ‘*’ in (**b**).

The purpose of this experiment was to evaluate our technique in an *in-vivo* setting involving relatively simple and electrically conductive biological tissues – a human calf. Figure 3a illustrates the experiment setup, with electrodes placed laterally on both sides of the left calf. Using the same experimental paradigm as the Phantom experiment, current-induced magnetic fields along *B*_z_ were measured for ‘Active’ and ‘Sham’ sessions respectively (Fig. 3b,c, thresholded to p < 0.05, Cluster corrected α < 0.05). In the ‘Active’ session, magnetic fields close to the cathode electrode were observed to decrease with current while an increase of magnetic fields was observed on the ventral calf. No significant induced fields were detected for the ‘Sham’ session. Figure 3d shows corresponding scatter plots for the two clusters (identified with an asterisk ‘*’ in Fig. 3b). Measured current-induced fields were strongly correlated with the applied current in both clusters, with the magnitude of induced fields per unit applied-current in the range of 30–45 nT/mA.

**Figure 4:**
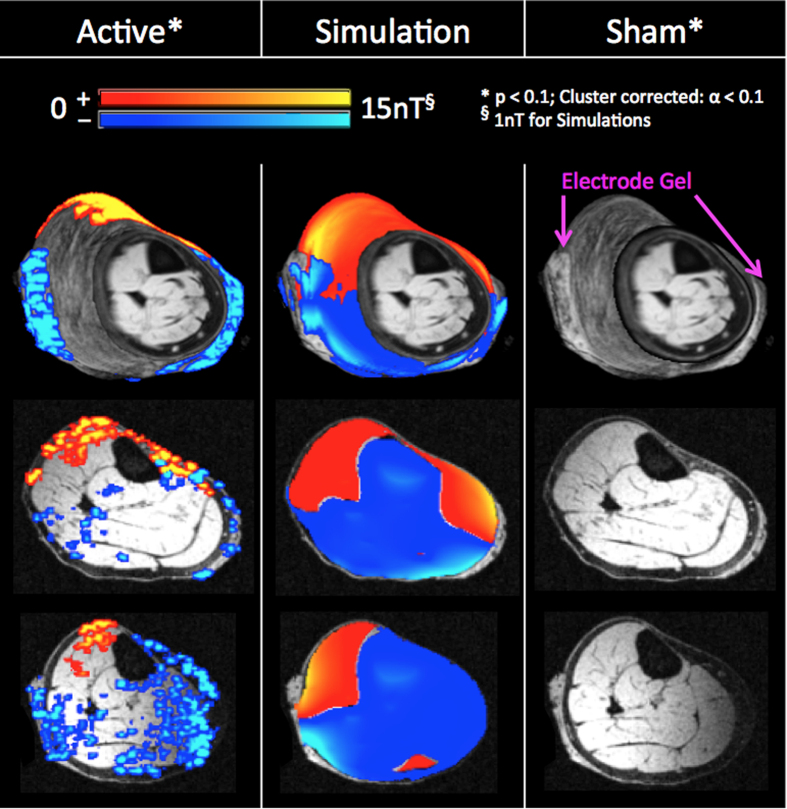
Comparison of the experimental results with simulated current-induced magnetic fields in a human calf. Column 1 shows the experimental results from the ‘Active’ session, which matches qualitatively well with the simulations in Column 2 (Cross-voxel Spearman correlations between the slices shown for simulated and ‘Active’ were r = 0.43, p = 2.4 × 10^−94^; N = 2072 (middle row) and r = 0.20, p = 1.5 × 10^−42^; N = 4571 (bottom row). No significant fields were detected for the ‘Sham’ session (Column 3).

We further compared the *in-vivo* results with simulated tDCS induced magnetic fields. The simulations were based on a finite element computational model of the human calf, utilizing tissue conductivity values from literature. In order to show the match between our experimental results and the simulated magnetic fields that are smooth in space, we had to apply a more liberal threshold of statistical significance for our experimental data (p < 0.1 and Cluster corrected at α < 0.1, Fig. 4). As can be seen in Fig. 4, the *in-vivo* detected magnetic fields match qualitatively well with the theoretically predicted magnetic fields in terms of their polarity, but show variations in spatial distribution (Spearman correlation between slices shown in Fig. 4: r = 0.43; p = 2.4 × 10^−94^; N = 2072 (middle row) and r = 0.20; p = 1.5 × 10^−42^; N = 4571 (bottom row)). Additionally, the magnitude of *in-vivo* detected magnetic fields was greater than those of simulated fields (which will be discussed below). No significant tDCS induced magnetic fields were detected for the ‘Sham’ session even with the more liberal threshold.

### Head Experiment

**Figure 5:**
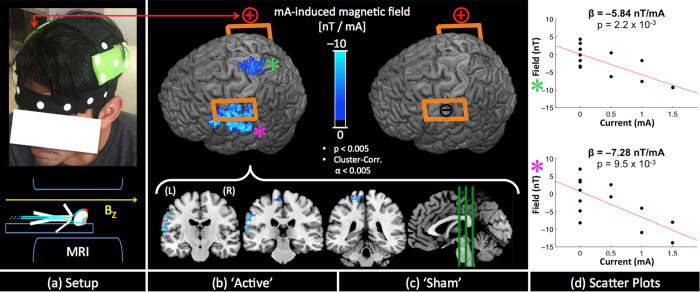
Head Experiment setup and results: (**a**) experimental setup with tDCS electrodes placed over motor cortices bilaterally; (**b**) significant (p < 0.05, Cluster corrected α < 0.05) results consistent across the group for the ‘Active’ session are (cluster size, MNI coordinates, peak in nT/mA): (575, [−60 16 26], −12.13) for left central sulcus and (161, [−4 −56 56], −7.33) for precuneus; (**c**) No significant results were observed for the ‘Sham’ session; (**d**) scatter plots of the two significant clusters identified with an asterisk ‘*’ in (**b**).

Finally, we applied our technique on healthy volunteers receiving tDCS according to a common tDCS montage and standard stimulation parameters, with the anode placed over the right motor cortex and cathode over the left motor cortex (C4/C3, based on the 10–20 system) (Fig. 5a). The purpose of this experiment was to demonstrate the validity of the proposed technique for a commonly used tDCS setup. Using an experimental paradigm identical to the limb and phantom experiments, MRI field mapping data were collected for ‘Active’ and ‘Sham’ sessions (counterbalanced across 12 healthy subjects). Group level analyses identified consistently reduced magnetic fields (along *B*_*z*_) at the left central sulcus under the cathode and in the precuneus region between the cathode and the anode electrodes (Fig. 5b). No significant induced magnetic fields were observed for the ‘Sham’ session (Fig. 5c). Scatter plots (Fig. 5d) show the average induced fields as a function of applied current for the two significant regions. Measured current-induced fields were negatively correlated with applied current, with the magnitude of the induced fields per unit applied-current in the range of 5–8 nT/mA. Note the wider spread of data points at 0 mA (also seen in the calf and phantom scatter plots) is due to the fact that there were 7 measurements at 0 mA versus 2 measurements at 0.5, 1 and 1.5 mA respectively.

Although voxel-wise group analysis did not show significant magnetic field changes under or around the anode electrode in the ‘Active’ session, region-of-interest (ROI) analysis based on the cortical location of C3/C4 electrodes^23^ revealed significant magnetic field reductions with applied tDCS current under both electrodes (–6.1 nT/mA, –4.4 nT/mA; p = 0.036, 0.044; for C3/C4 respectively, N = 12 subjects, see Supplemental Fig. S1). No such fields were detected for the ‘Sham’ session.

## Discussion

### Feasibility and Validity of the Proposed Technique

The key innovation of our technique is that it provides quantification of magnetic fields directly induced by tDCS currents as a means to visualize target engagement. This is in contrast to present *in-vivo* imaging approaches that use surrogate markers to record the brain’s neurovascular and neurophysiological responses to tDCS stimulation (e.g., BOLD/ASL fMRI, EEG and MEG). Proof of concept was established through the phantom experiment where the measured current-induced magnetic field was in excellent agreement with simulations (r = 0.84, p = 3.8 × 10^−267^, N = 989). The phantom results also demonstrated excellent specificity as no significant magnetic fields were detected in either the within-session control (Tube ‘B’), or during the control session (‘Sham’). In its present implementation, our technique is capable of detecting fields as low as one nT per mA of applied current (Supplemental S1) with a spatial resolution of a few millimeters.

The limb experiment demonstrated *in-vivo* feasibility of our technique in a relatively simple and electrically conductive biological tissue. While no field changes were detected during the ‘Sham’ session, magnetic field changes greater than 30nT/mA were observed during the ‘Active’ session. The experimental results also qualitatively matched the theoretically predicted magnetic fields based on a finite element model of the human calf (r = 0.43, p = 2.4 × 10^−94^; N = 2072). However, unlike the phantom experiment the experimentally detected magnetic fields were found to be greater than the simulated fields. This difference could be due to heterogeneity in the tissue conductivity leading to potential “hotspots” of electromagnetic fields that were detected by *in-vivo* experiment but not theoretical modeling. Another explanation could be variations in the tissue conductivity as well as boundary conditions employed by the computational modeling. These observations highlight the need for reliable *in-vivo* mapping of electromagnetic fields even for organs and limbs with relatively simple biological compositions.

Finally we performed the brain experiment with a common tDCS montage (bilateral-M1) on 12 healthy volunteers. A group level analysis revealed relatively weak (5–8nT/mA) yet statistically significant magnetic field reductions around the central sulcus (underneath the cathode) as well as in the precuneus region (around mid-way between the electrodes). Our observations are consistent with modeling studies which have predicted peak current densities to exist under the electrodes^24^, and for large electrodes (25–35 cm^2^) less than 10 cm apart, a single peak current density to exist between the electrodes^25^. Moreover, the magnitude of the experimentally detected fields, 0–10 nT/mA, was found to be the same order as that of simulated fields reported in a recent study^21^ (although the study used a different cathode positioning). Furthermore, although significant field changes were not detected under or around the anode in voxel-wise group analysis, an ROI analysis (Supplemental Fig. S1 with the method described in Supplemental S5) revealed significant field changes under both electrodes (and none during Sham). These field changes were also observed to have the same sign; which is intuitive given that the direction of tDCS current flow is the same (from anode to cathode) at both electrodes. However, it is not clear why the field changes were more significant under cathode than anode in our experiment. One potential explanation is the relatively large electrode size making the induced electromagnetic fields “diffuse”, and such effect may be asymmetric between anode and cathode. With improved sensitivity of our technique (see below) and improved focality of tDCS montage (e.g., high-definition tDCS)^20^, we will be able to evaluate such hypothesis in future studies.

### Advantages of the Proposed Technique

The proposed approach for visualizing tDCS target engagement through its induced magnetic fields is appealing since the magnetic field is directly induced by and linearly proportional to the applied current (as described by Ampere’s law). Moreover, the physical constant involved in the relationship between magnetic field and direct current is highly stable across biological tissues (variation of magnetic permeability is on the order of ~ppm^26^). In contrast, existing experimental techniques use surrogate markers of the brain’s response to tDCS stimulation (e.g., BOLD, ASL, EEG, MEG). These represent a secondary response to the applied stimulation, and may extend beyond the site of stimulation due to brain networks. Additionally, unlike the physical relationship between magnetic fields and applied current, the relationship between these markers and the applied tDCS current is highly complicated, and not easy to interpret.

In applications involving milliampere currents, the primary challenge for detecting current-induced magnetic fields is the weak signal relative to noise (SNR). Existing techniques to detect weak magnetic fields attempt to overcome the SNR limitation primarily through enhancing the ‘signal’, e.g., by increasing the current intensity and/or using time varying currents (~1 Hz or higher)^27^,^28^,^29^. In contrast, our technique addresses the SNR limitation by statistically modeling out a range of ‘noise’ sources by exploiting the linear relationship between applied-current and induced magnetic fields, and is thus able to detect small magnetic fields with high sensitivity and spatial resolution.

In the proposed technique, a general linear model (GLM) was employed to model a set of magnetic field maps with applied current as well as systematic noise sources, similar to linear regression analysis commonly used in fMRI. Our choice of a GLM is supported by the fact that the relationship between applied current and measured magnetic field is linear (Ampere’s Law), and by ensuring the stochastic-noise distribution is Gaussian (see Supplemental S5, ‘Preprocessing’). Another advantage of using GLM is that many statistical methods developed for fMRI can be adapted for the proposed technique. For instance, in an experimental paradigm involving tasks, tDCS induced signals could be separated from task-related brain signals^30^ by designing the tDCS and task stimuli to be sufficiently orthogonal (similar to optimal stimulus design in fMRI). In one possible implementation, a study could involve two identical task sessions: one with ‘Active’ tDCS currents, and another with ‘Sham’. The contrast between ‘Active’ and ‘Sham’ sessions would detect magnetic field changes specifically induced by tDCS, controlling for task related brain-activity signals. The use of a GLM does assume (implicitly) the invariance of current path with different intensities of applied currents. This assumption is reasonable considering the small magnitude of currents used (will not induce neuronal discharge) and the fact that all currents were applied in the same direction.

### Potential Clinical Applications

In the present study, we were able to visualize tDCS current-induced magnetic fields in human brains at the group level. This is a significant first step to experimental verification of target engagement. In addition to verification, our technique can also help advance focal stimulation, which has been shown to be more efficient than conventional tDCS^31^. By identifying group-wide peak-intensity areas from imaging studies (performed for conventional tDCS), our technique can potentially (a) provide target engagement confirmation for a conventional tDCS trial; and if treatment effects are observed, (b) identify target sites for future studies with focal stimulations with a reasonable expectation of the same treatment effects. Once we reach the capability for reliable mapping of electromagnetic fields induced by tDCS in individual subjects with the proposed technical developments (see below), our technique may open the door to a new field of individualized, precise, noninvasive neuromodulation using tDCS.

### Limitations and Future Developments

With the present implementation (single-channel coil and standard field mapping sequence), we were able to reliably detect significant field changes during the head experiment at the group level but not in individual subjects. To address this shortcoming, future studies will use faster field mapping sequences to increase the number of measurements per session (thereby increasing statistical power) together with multi channel coils to improve SNR. The increased SNR may also improve the sensitivity of the proposed technique (~1.2 nT/mA of the present implementation, see Supplemental S1). The capability to reliably map tDCS current-induced fields in individual subjects may also overcome the potential averaging effects of group analysis, given the variability of cortical geometry and current distribution across subjects, and allow direct comparison with computational models.

It should be noted that our present technique maps only the magnetic field changes along *B*_*z*_. In the absence of additional information, mapping electric current requires measuring all three spatial components of the induced field. One possible solution is to map the induced fields of a subject in at least three different orientations, which may be feasible with an open magnet. Alternatively, constraining computational current prediction models with a single experimentally verified component of the induced field could improve the accuracy for predicting current distribution.

A promising direction for research involves integrating fMRI with our field mapping technique. While our technique uses the phase information in an MRI image, most existing fMRI methods use magnitude information. Since every MRI acquisition generates phase and magnitude images, it is conceivable that target engagement (through mapping tDCS induced magnetic fields) and ensuing neurophysiological effects (through BOLD/ASL fMRI) could be simultaneously measured during a single tDCS session. One such promising technique is ASL with dual-echo EPI^32^ readout to simultaneously map current induced fields, perfusion and blood oxygenation.

## Methods

### Theory

#### “Electrical current induces Magnetic Fields”

In classical electrodynamics, Maxwell’s equations describe the generation and interaction of electrical and magnetic fields. In particular, Ampere’s law (Equation (1)) describes that an applied electrical current induces a magnetic field


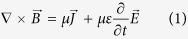


Here, 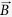 is the induced magnetic field, 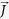 is applied tDCS current density, *t* is time, *μ, ε* are physical constants (magnetic permeability and electrical permittivity respectively, of biological tissue), 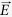 is the electrical field and 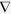 is the curl operator (details provided in Supplemental S6). For direct current, as is the case of tDCS, the electric fields do not vary with time making the last term *δ*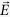/*δt* zero. Consequently, the induced magnetic field is directly proportional to the applied tDCS current density (along an orthogonal direction).

#### “MRI detects induced magnetic fields”

Magnetic resonance imaging (MRI) is able to map changes in magnetic field along the static magnetic field (*B*_z_). Field disturbances perpendicular to *B*_z_ are generally invisible to MRI, as a consequence of the fact that (a) the *B*_z_ magnetic field is orders of magnitude larger than any typical disturbance and (b) magnetic fields add vectorially (Supplemental S2). Using MRI field mapping, field variations along *B*_z_ can be measured as phase angles according to


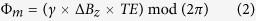


where *Φ*_m_ is the measured phase angle between 0 and 2π radians, *γ* is gyromagnetic ratio (a constant), Δ*B*_z_ is the field deviation along *B*_z_ and *TE* is the echo time. A practical aspect of phase measurement is that phase angles outside (0, 2π) are mapped back onto this range causing a ‘wrap’ (represented by the modulo operation in Equation (2)). An acquired phase-map has to be unwrapped to calculate the correct phase to infer the underlying field.

### Simulation

Simulations were employed to validate the proposed technique. Our first experiment involved a phantom specifically designed to confine the applied current to a known path. This enabled the calculation of the current-induced magnetic field using the Biot-Savart law:


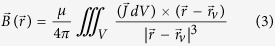


where 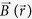 is the magnetic field at the position 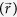, *μ* is the magnetic permeability, 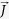 and 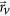 are the current density and position vectors respectively of the finite volume element *dV*, and the integral sum is over the entire volume *V*. In the case of our second experiment (calf), the currents were calculated using a finite element model (FEM) solver. The implementation details of the finite element simulations are provided in Supplemental S3 (Phantom) and Supplemental S4 (Calf).

### Experimental Design

All data were acquired on Siemens 3T MR scanners using single-channel Tx/Rx coils and a custom built MRI-compatible tDCS system. *In-vivo* data were acquired from 13 healthy participants (1M, 36 yrs, calf; 7M/5F, 29.7 ± 8.2 yrs, head). Subjects were screened for neurological/psychiatric disorders. Written informed consent was obtained from all participants. The study was approved by the Institutional Review Board (IRB) at the University of California Los Angeles. All experiments and data acquisition were performed in accordance with the guidelines and regulations set by the Institutional Review Board (IRB) at the University of California Los Angeles.

**Figure 6:**
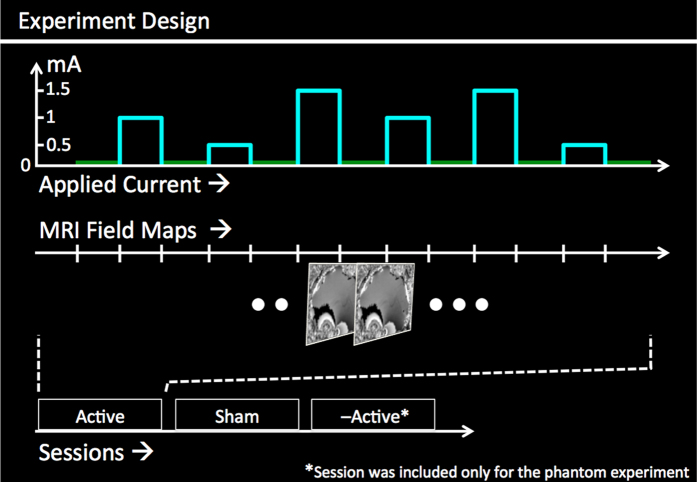
Experiment design: Data were collected from 2 sessions: ‘Active’ and ‘Sham’. The order of the sessions was counterbalanced across subjects for head experiment. Each session consisted of 6 currents applied in pseudorandom order with 7 interleaving 0 mA currents. Field maps were acquired for each applied current. An additional ‘–Active’ session was included for the Phantom experiment, wherein the polarity of the applied currents was reversed (compared to ‘Active’).

Each concurrent tDCS-MRI experiment consisted of two back-to-back sessions (‘Active’ and ‘Sham’) with a single-blinded design. The order of these sessions was counterbalanced across subjects for the head experiment. Each session consisted of 6 currents (and corresponding field mapping scans) applied in a pseudo random order interleaved with zero-current scans (a total of 13 field mapping scans per session, Fig. 6). Before each field mapping acquisition, current was ramped up and maintained for 20 sec. During the ‘Sham’ session, currents were ramped down back to zero before the MRI scan. In the Phantom experiment, an additional ‘–Active’ session was included where the direction of the applied currents was reversed. In all other aspects, ‘Sham’ and ‘–Active’ sessions were identical to ‘Active’.

Field mapping data was acquired using a dual-echo gradient-echo sequence with the following parameters: TE_1_/TE_2_ = 4.92/14.76 msec, TR = 1.15 sec, Matrix: 128 × 128, BW = 750 Hz/pix, FA = 25°, 65 slices, 2 ×2 ×3 mm^3^ Voxel. For the calf experiment, the flip angle, total slices and voxel size were adjusted to 65°, 55 and 1.2 × 1.2 × 3 mm^3^ respectively. Both magnitude and phase data were acquired from field mapping scans. A T1-weighted MPRAGE structural scan (TE_1_ = 4.82 msec, TI = 1100 msec, TR = 2.17 sec, Matrix: 256 × 256, BW = 140 Hz/pix, FA = 7°, 192 slices, 1 × 1 × 1 mm^3^) was also acquired.

### General Linear Model Analysis

#### Measured phase was modeled as:


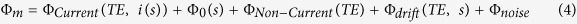


where *Φ*_m_ is the measured phase, *Φ*_Current_ is the phase due to current-induced fields, *TE* is the echo-time, “*s*” refers to the fact that the data is from the *s*^th^ scan of the session and *i*(*s*) is the current applied during the “*s*-th” scan. *Φ*_0_ is the baseline phase, *Φ*_Non-Current_ is the phase due to field deviations unrelated to applied current but steady between scans (e.g., off-resonance), *Φ*_drift_ is the phase due to inter-scan field-deviations caused by the time-varying drift of the main magnetic field and *Φ*_noise_ is the phase due to (Gaussian) noise.

Acquired phase data were fit to the applied current within the framework of a GLM. The slope of this fit was converted to the induced magnetic field per unit mA applied-current using Equation (2). Additional details of the concurrent tDCS MRI system, data acquisition, data processing, modeling (including that of confounds) and subsequent statistical analysis are discussed in detail in Supplemental S5. By incorporating experimental parameters and hardware constraints (*TE* and minimum detectable phase-angle respectively) within Equation (2), the theoretical detection limit of our technique in the present implementation was determined to be ~1.2nT/mA (Supplemental S1).

## Additional Information

**How to cite this article**: Jog, M. V. *et al*. In-vivo Imaging of Magnetic Fields Induced by Transcranial Direct Current Stimulation (tDCS) in Human Brain using MRI. *Sci. Rep.* **6**, 34385; doi: 10.1038/srep34385 (2016).

## Supplementary Material

Supplementary Information
