# Supplementary material for: In-vivo Imaging of Magnetic Fields Induced by Transcranial Direct Current Stimulation (tDCS) in Human Brain using MRI
Source: Sci Rep. 2016 Oct 4;6:34385. doi: 10.1038/srep34385 (PMC5048181; doi:10.1038/srep34385)
Supplement: Supplementary Information [file srep34385-s1.pdf]

Supplemental Materials Online

In-vivo Imaging of Magnetic Fields Induced by Transcranial Direct Current Stimulation (tDCS) in Human Brain using MRI

Mayank V Jog, Robert X Smith, Kay Jann, Walter Dunn, Belen Lafon, Dennis Truong, Allan Wu, Lucas Parra, Marom Bikson, Danny JJ Wang*

S1: Detection Limit of the Proposed Technique

Equation (2) in main text can be rewritten as:

$$\Delta B_z = \frac{\Delta \Phi_m}{\gamma \times \Delta TE} \quad \text{(S1)}$$

where the quantities are as those defined in ***Equation (2)***, and Δ represents the fact that we are considering the phase difference between two TE's (The phase difference is utilized in actual analyses, since it removes a confound, see *Supplemental S5*). The modulo-2π operation has been removed reflecting the fact that the phase angles have been unwrapped. In an ideal scenario without noise, the minimum (unbiased) detectable field corresponds to the smallest possible non-zero phase change generated by the smallest applied current (0.5mA). Because the full phase range of 0 to 2π radians is divided into 4096 discrete levels in MRI, the smallest non-zero phase change evaluates to $\left(\frac{2\pi}{4096}\right)$ radians. Together with the experimental parameter ΔTE (chosen as 9.84 msec for all our experiments), the minimum (unbiased) detectable field equates to 0.58nT at 0.5mA, or ~ 1.2nT/mA. It should be noted that this detection limit is specific to the present implementation. In other words, it is possible to lower this limit using a larger ΔTE, and to some extent, different applied currents. A larger ΔTE can be achieved by minimizing TE_1, or, increasing TE_2 permitted by SNR.

S2: MRI is only sensitive to magnetic field disturbances along B_z

Here we show why MRI is only sensitive to magnetic field disturbances along the static magnetic field (B_z). Any perturbation of the magnetic field ($\Delta\vec{B}$) can be written as a sum of two orthogonal components: one along B_z, and the other perpendicular to B_z (indicated by $(\Delta B_{\parallel})\,\widehat{B_z}$ and $(\Delta B_{\perp})\,\widehat{B_{\perp,z}}$ respectively).

The total magnetic field can be written as a vector sum of B_z and the perturbation:

$$\vec{B}_{total} = (B_z + \Delta B_{\parallel})\,\widehat{B_z} + (\Delta B_{\perp})\,\widehat{B_{\perp,z}} \qquad \text{(S2)}$$

or equivalently, as a vector of length B_{mag}, at an angle θ to $\widehat{B_z}$:

$$B_{mag} = \sqrt{(B_z + \Delta B_{\parallel})^2 + (\Delta B_{\perp})^2} \quad ; \quad \theta = \tan^{-1}\left(\frac{\Delta B_{\perp}}{B_z + \Delta B_{\parallel}}\right) \qquad \text{(S3)}$$

For perturbations on the order of ppm, it can be seen that θ is almost zero i.e., $\vec{B}_{total}$ is along $\widehat{B_z}$. Under the same ppm perturbations, B_{mag} can be approximated using the Taylor Series expansion as

$$B_{mag} \cong B_z + \Delta B_{\parallel} + \frac{B_z}{2}\left\{\left(\frac{\Delta B_{\parallel}}{B_z}\right)^2 + \left(\frac{\Delta B_{\perp}}{B_z}\right)^2\right\} + \ldots \qquad \text{(S4)}$$

Relative to $\Delta B_{\parallel}$, the contribution of $\Delta B_{\perp}$ is on the order of ppm (since it is scaled by a factor of B_z^{-1}) and can thus be neglected. Thus, it can be said that (1) the magnetic field deviations measured by MRI field mapping reflect $\Delta B_{\parallel}$, i.e. the component of the field disturbances along $\widehat{B_z}$ and (2) the resultant field is along the MRI static field ($\because\ \theta \approx 0$).

S3: Simulations of Phantom Experiment

In the phantom experiment, the current density at each point of the current carrying tube can be

estimated. In such a special case involving known DC current densities, the Biot-Savart law can be used to calculate current-induced magnetic fields (***Equation (3)*** in main text).

An implementation of the Biot-Savart law for line currents (33) was modified in-house and extended to volumetric finite element currents. The implementation of the finite element simulation was carried out in two steps:

1. Magnetic field estimation:

   Magnetic fields (along B_z) were estimated over a grid (henceforth referred to as the 'Sampled grid', *Supplemental Fig. S2(a)*) designed to match the spatial resolution of the MRI phantom experiment $\Delta x \times \Delta y \times \Delta z = 2 \times 2 \times 3\ mm^3$. It should be noted that while MRI measurements represent the average magnetic field in a voxel, magnetic fields calculated using the Biot-Savart law estimate the field at a point and hold no information about the neighborhood. To address this, the magnetic field at each point on the sampled grid was calculated as an average over fields calculated on a 3D 'super-sampled' grid (*Supplemental Fig. S2(b)*, shown as 2D for simplicity). The spatial resolution of the 3D super-sampled grid was 0.5 mm isotropic and the magnetic field value at each point was calculated using the Biot-Savart Law (***Equation (3)*** in main text).

2. Electric Current modeling:

   Assuming the salt-water electrolyte to be isotropic with uniform conductivity, the electric current is expected to travel in parallel 'streamlines' between the electrodes. Utilizing the inherent symmetries in the system, these streamlines were discretized to a non-Cartesian grid as shown in *Supplemental Fig. S2(c)*. The discretized grid had a spatial resolution of

~0.125mm isotropic. In other words, at least 64 points would be enclosed by a volume with the size of a single voxel on the super-sampled grid (discussed in 1 above).

Each streamline is an electric current flowing through an ohmic resistor. Thus, the system is electrically equivalent to a current-divider circuit (shown in *Supplemental Fig. S2(d)*), with each branch of the circuit representing a streamline and the input being the total applied current. The resistance of each branch is equal to the resistivity of the electrolyte multiplied by the ratio of the length of the streamline to its cross-sectional area. While the length of a particular streamline is known from phantom geometry, the cross-sectional area is determined during the discretization process. The circuit was solved to compute the current through each streamline by choosing the total applied current to be 1mA. It should be noted that the resistivity of the electrolyte is not needed for this calculation. The choice of 1mA enabled direct comparison between the simulated fields and those detected using MRI (the latter being the induced magnetic fields per unit mA applied-current). The average current density within the conducting tube 'A' was 0.79 mA/cm^2 (1mA, ½ inch tube diameter).

S4: Simulations of Limb Experiment (Calf)

Laplace's equation governs the voltage distribution in a purely resistive volume during constant current flow. The calf structural MR images were segmented into compartments representing the gel, skin, fat, muscle and bone using a combination of automated and manual segmentation routines (17). The stimulation electrodes were imported as CAD models and positioned within the image data. Volumetric meshes were subsequently generated from the compartments (SIMPLEWARE Ltd, Exeter, UK) and imported to a commercial finite element solver

(COMSOL Inc, MA, USA). Isotropic conductivity values were used (electrode: 5.99e7, gel: 1.4 S/m, skin: 0.465, fat: 0.001, muscle: 0.16, bone: 0.01) and the Laplace equation was solved to generate a spatial map of current density. The conductivity values used have been previously reported in (34) (skin and bone) and (35)(muscle and fat).

The Biot-Savart law (***Equation 3*** in main text) was used to calculate magnetic field due the calculated static current densities. The Biot-Savart law was implemented as a 3D convolution, as previously reported in (21).

S5: Experimental Procedures

Concurrent tDCS-MRI

An MRI compatible tDCS system was constructed to enable concurrent tDCS-MRI experiments. Quad-shielded coaxial cables were used to carry the applied current from the tDCS stimulator (kept in the MRI control room) to the electrodes (in the scanner room). The battery-powered "ActivaDose Iontophoresis Delivery Unit" (ActivaTek, Salt Lake City, UT) was used to drive the current. Similar to (13), a filter box and in-line resistors of 10k-ohm were installed at the stimulator and electrode ends respectively to provide protection from potential voltage fluctuations due to gradient switching during MRI scans.

Experiments

A. Phantom:

The phantom was constructed in three parts –

(i) Bulk phantom: A standard Siemens cylindrical phantom (3.75g $NiSO_4$ x $6H_20$ + 5g NaCl per

1000gm H_2O, 0.5 Gallon, 10.6 cm diameter) was used. Available in a sealed plastic container, the contents are insulated from external currents.

(ii) Current-Carrying tube (Tube 'A', **Fig. 1a**): A flexible plastic tube (ID=1/2 inch) was bent into a U-shape and wrapped around the long axis of (i) at its midline. The tube was filled with salt water (electrolyte), and fitted with copper electrodes at both ends. The electrodes were connected to the tDCS stimulator. All applied currents travel through this tube.

(iii) 'Control' Tube (Tube 'B', **Fig. 1a**): A second plastic tube (ID=3/8 inch) constructed using the same material and electrolytes, was placed similar to (ii) at the distal end of (i). This tube was designed to serve as an intra-session control and accordingly, the electrodes were not connected to the tDCS stimulator.

B. Calf:

Round carbon rubber electrodes (2" inch diameter) were secured laterally on each side of the left calf. Conductivity gel (Cadwell, P/N# 202153-000) was used to make electrical contacts.

C. Head:

Sponge electrodes were placed bilaterally over the C3/C4 position (anode C4 and cathode C3), according to the international 10/20 system to target the motor cortices. The electrodes were rectangular (4.5x9cm) in shape with their long axes in A←→P direction. The same conductivity gel as the calf experiment (Cadwell, P/N# 202153-000) was used to make electrical contacts.

Data Acquisition

MRI data were acquired using a product single channel Tx/Rx quadrature volume coil. The

phantom and calf data were acquired on a Siemens 3T PRISMA system, while the head data were acquired on a Siemens 3T Trio TIM system. It should be noted that MRI shimming was performed only once at the start of each session. Shimming is a pre-scan preparatory step that corrects for field deviations from B_z and may cancel out current induced fields. Potentially uncorrected field deviations, unrelated to electrical current, were explicitly modeled out (see Φ_{drift} below).

Pre-Processing

Stochastic noise in a single voxel of the phase image is approximately zero-mean Gaussian for (magnitude) SNR >=3 (36). A Gaussian noise is necessary for phase unwrapping as well as for GLM modeling of the preprocessed data (especially since at the other extreme of SNR=0, voxel noise in the phase image is uniformly distributed). The threshold of 3 is mathematically equivalent to a p-value (since the distribution of the noise is known). This p-value threshold was adjusted (Bonferroni corrected) to account for the total number of voxels being tested, and voxels under the threshold were excluded.

Thresholded phase data was unwrapped using the Region growing algorithm implemented in the PhaseTools toobox (37) (available as a plug-in for Fiji (38)). Unwrapping was carried out at the individual volume level, followed by unwrapping within each slice. Using SPM8, volumes were then realigned to the first (volume) to account for inter-scan motion. The realignment parameters were estimated using the magnitude data from the first echo (TE_1). The realignment step was skipped for the phantom.

Model for Measured Phase

Measured phase was modeled as:

$$\Phi_m = \Phi_{Current}\big(TE, i(s)\big) + \Phi_0(s) + \Phi_{Non-Current}(TE) + \Phi_{drift}(TE, s) + \Phi_{noise} \qquad \text{(S5)}$$

where Φ_{m} is the measured phase, Φ_{Current} is the phase due to current-induced fields, *TE* is the echo-time, "*s*" refers to the fact that the data is from the sth scan in the current session and $i(s)$ is the current applied during the "*s*-th" scan. Φ_0 is the baseline phase, $\Phi_{\mathrm{Non\text{-}Current}}$ is the phase due to field deviations unrelated to applied current but steady between scans (eg. off-resonance), Φ_{drift} is the phase due to inter-scan field-deviations caused by the time-varying drift of the main magnetic field and Φ_{noise} is the phase due to (Gaussian) noise.

The phase difference ($\Delta\Phi_{\mathrm{m}}$) between the two TEs was computed. This step eliminates Φ_0. $\Delta\Phi_{\mathrm{m}}$ was subsequently included as the dependent variable in a general linear model (GLM) with applied current ($i(s)$) as the predictor. This is based on the assumption that the current path remains the same for all applied currents. $\Delta\Phi_{\mathrm{Non\text{-}Current}}$ by definition does not vary with applied current and is implicitly incorporated into the GLM intercept.

On the other hand, $\Delta\Phi_{drift}$ was explicitly modeled by a polynomial function that was fitted least squares wise to phase measured during zero-current scans. This is consistent with the model for measured phase (***Equation (S5)***) wherein the phase fluctuations for zero-current scans should be purely due to drift and noise. The degree (d*) of the polynomial was selected to optimally match the characteristics of the residual with those of Φ_{noise} to prevent overfitting. Φ_{noise} for each voxel was estimated using the magnitude image (36).

The regression coefficient for applied current (β_{Current}) obtained from the GLM analysis can be interpreted as the phase gained per 1mA applied-current. This was converted to induced magnetic field per mA current using ***Equation (2)***. Obtained mA-current induced magnetic field maps were subsequently thresholded at $p<0.05$ and cluster corrected (using AlphaSim (39)) following standard statistical procedures for fMRI.

Group Level Analyses

Current-induced field maps of individual subjects were coregistered to the corresponding structural images and subsequently normalized to the MNI space using SPM8. Normalized field maps were used to perform a one-sample t-test for both 'Active' and 'Sham' sessions. Results were thresholded at $p<0.05$ and cluster corrected for multiple comparisons using AlphaSim (39).

Region-of-interest (ROI)-Analysis

ROIs were constructed as spheres with a radius of 1cm centered at projections of C3/C4 on the cortex (as reported by (23)), thresholded to exclude all out of brain voxels. Each ROI enclosed equal number of voxels (178). $\Delta\Phi_{\mathrm{Current}}$ within each ROI was averaged over all voxels, and was used in a linear fit with applied current as the predictor. The slope of such a fit can be interpreted as the ROI-averaged current-induced field. These were subsequently used to perform a one-sample t-test for both 'Active' and 'Sham' sessions. Significant current induced magnetic fields were observed for the 'Active' session, both at C3 and C4 (corresponding to Cathode and Anode respectively). No fields were observed for the 'Sham' session (See *Supplemental Fig. S1*). Additionally, the field changes under anode and cathode had the same sign; which is intuitive

given that the direction of tDCS current flow is the same (from anode to cathode) at both electrodes. Note that the sign of the fields matches that of the results obtained through voxel-wise analysis.

S6: Ampere's Law for Biological Tissues

The general form of Ampere's Law is:

$$\nabla \times \vec{H} = \vec{J} + \frac{\delta}{\delta t}\vec{D} \quad \text{(S6)}$$

where $\vec{H}$ is the magnetizing field, $\vec{J}$ is the applied current, $\vec{D}$ is the displacement field, t is time and ∇ is the curl operator. The time-varying term can be ignored for our DC case. Our experiment consists of applying a current to biological tissues, which induces magnetic field perturbations ≤ μT around a 3T static field (B_0) of the MRI. Under these conditions, it is appropriate to replace $\vec{H}$ according to

$$\vec{B} = \mu\vec{H} \quad \text{(S7)}$$

where $\vec{B}$ is the induced magnetic field, and μ is the magnetic permeability of the biological tissue (26). Under the same conditions, μ is reported to be a scalar, varying on the order of ppm between tissues (26). By using ***Equation (S7)*** in ***Equation (S6)***, ***Equation (1)*** in main text can be derived. A key point to note is that for the reported tissue μ, the relationship between applied current $\vec{J}$ and induced magnetic field $\vec{B}$ is linear. Note that the linearity relationship holds even if μ is a tensor. In fact, the independence of μ from $\vec{B}$ or $\vec{H}$ is a sufficient condition for linearity to hold.

Supplemental Figures

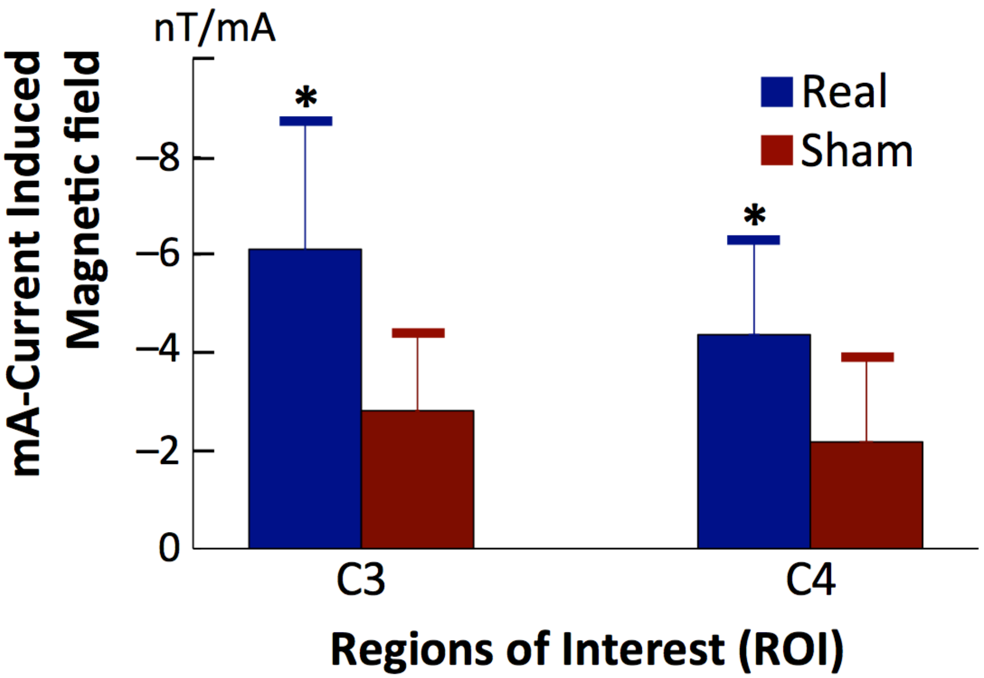

Figure S1: Results of one-sample t-test performed on ROI-averaged current-induced field. Significant current-induced magnetic field reductions were detected at the C3 and C4 ROIs for the ‘Active’ session (β = –6.1 nT/mA, –4.4 nT/mA; p = 0.036, 0.044 respectively; N = 12 subjects). No significant fields were detected for the ‘Sham’ session (β = –2.8 nT/mA, –2.2 nT/mA; p = 0.104, 0.233 respectively). Also shown are error bars for the one-sample t-tests. See <u>*Supplemental S5*</u> for detailed methods for ROI analysis.

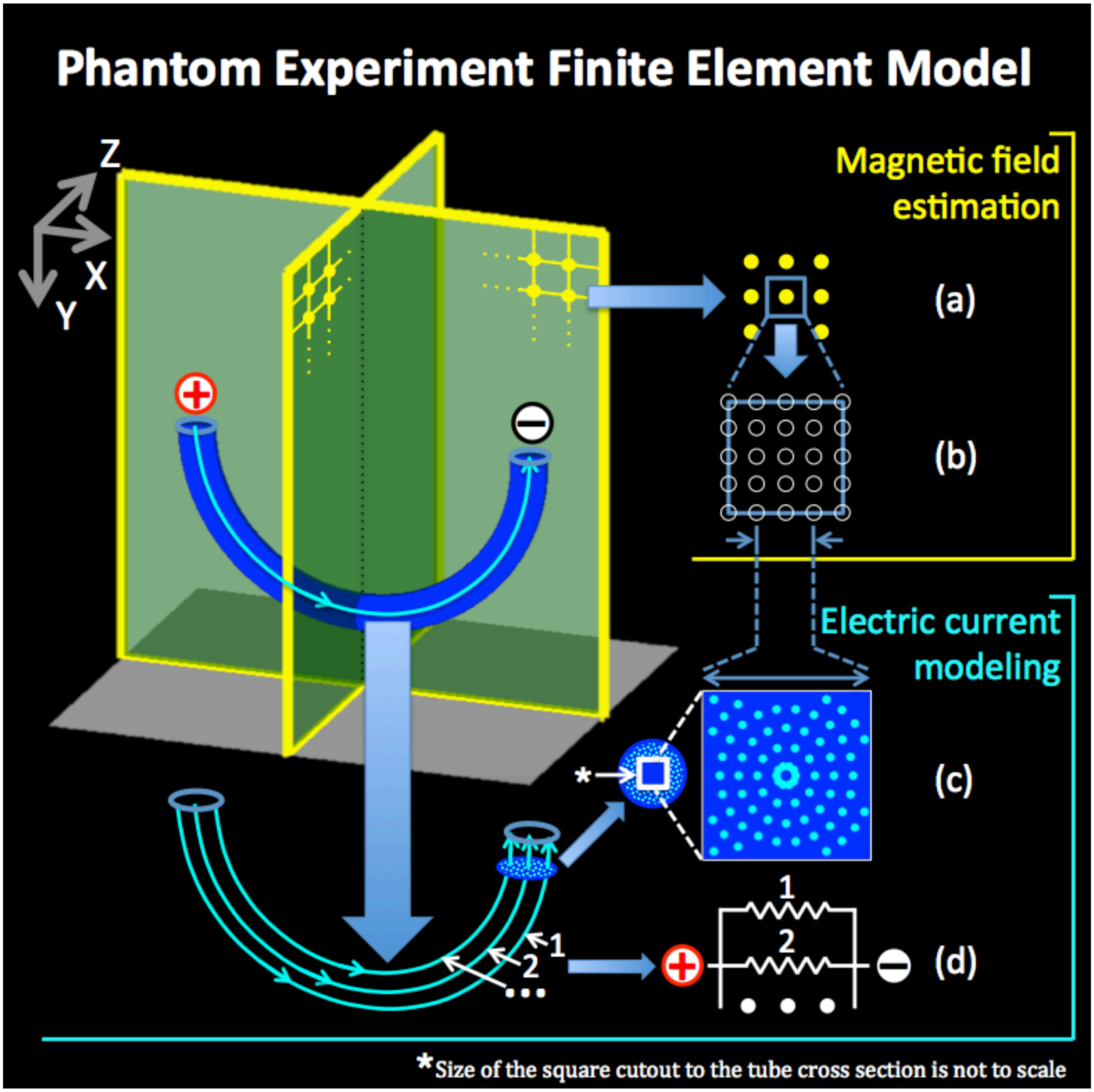

Figure S2: Finite Element modeling of the phantom experiment: (a) shows the grid where the magnetic field was calculated as an average over points on a 'super-sampled' grid (b), to emulate the fact that MRI measures the average behavior in a voxel. The applied current was modeled as streamlines, which were subsequently discretized as shown in (c). Current through each streamline was calculated from the equivalent circuit as depicted in (d)
